# Membrane-assisted extraction of monoterpenes: from *in silico* solvent screening towards biotechnological process application

**DOI:** 10.1098/rsos.172004

**Published:** 2018-04-18

**Authors:** L. Janoschek, L. Grozdev, S. Berensmeier

**Affiliations:** Bioseparation Engineering Group, Department of Mechanical Engineering, Technical University of Munich, Boltzmannstr. 15, 85748 Garching, Germany

**Keywords:** membrane extraction, hollow-fibre membrane contactor, COSMO-RS, monoterpene, carvone

## Abstract

This work focuses on the process development of membrane-assisted solvent extraction of hydrophobic compounds such as monoterpenes. Beginning with the choice of suitable solvents, quantum chemical calculations with the simulation tool COSMO-RS were carried out to predict the partition coefficient (log*P*) of (S)-(+)-carvone and terpinen-4-ol in various solvent–water systems and validated afterwards with experimental data. COSMO-RS results show good prediction accuracy for non-polar solvents such as n-hexane, ethyl acetate and n-heptane even in the presence of salts and glycerol in an aqueous medium. Based on the high log*P* value, n-heptane was chosen for the extraction of (S)-(+)-carvone in a lab-scale hollow-fibre membrane contactor. Two operation modes are investigated where experimental and theoretical mass transfer values, based on their related partition coefficients, were compared. In addition, the process is evaluated in terms of extraction efficiency and overall product recovery, and its biotechnological application potential is discussed. Our work demonstrates that the combination of *in silico* prediction by COSMO-RS with membrane-assisted extraction is a promising approach for the recovery of hydrophobic compounds from aqueous solutions.

## 1. Introduction

Terpenes are a large class of naturally occurring hydrocarbon compounds. They are mostly produced in plant cells as secondary metabolites and have vast structural variety and diverse properties [1]. Terpenes are based on condensed and cyclized isoprene monomers which are biosynthesized via the mevalonate (MVA) or the methyl-D-erythritol-4-phosphate (MEP) pathway. The number of isoprene units forming the terpene skeleton defines the different terpene subclasses e.g. mono-, di- and sesquiterpenes [1,2]. Not only monoterpenes in essential oils, such as limonene and carvone, are used widely in the flavour and fragrance industries, but also interest in their therapeutic and pharmaceutical applications has grown. Owing to their diverse structures and functional groups, many terpenes show promising bioactivity or have already been introduced in anti-cancer, anti-inflammatory and anti-infective treatment applications, some of which are reviewed in certain publications [3–6]. Terpenes are mostly derived directly from their natural sources, but recent advances have been made to produce them by semi-synthesis or biotechnologically in plant cell cultures or microbial hosts such as yeast, fungi or bacteria [6–8]. Extraction techniques, such as supercritical CO_2_ extraction, microwave-assisted extraction and other solid–liquid extraction methods, are the most common for isolating and purifying hydrophobic terpenes and other natural products from plant-derived raw material [9]. When biotechnologically produced, terpenes or terpene-containing hosts are present in an aqueous environment like a fermentation medium or bioconversion buffer. This makes liquid–liquid extraction with a water-immiscible acceptor phase the most favourable recovering technique. Liquid–liquid extraction enables product enrichment even at low solute concentrations and volume reduction, which is crucial in biotechnological processing. The extraction efficiency mainly depends on the partition coefficient and therefore on the right choice of solvent. Most common are hydrophobic organic solvents such as aliphatic or cyclic hydrocarbons, and also alternative solvents, such as ionic liquids (IL) and deep eutectic solvents (DES), might be applicable when a more sustainable process with reduced environmental impact, but still with high extraction efficiency is required [10–13]. However, with complex solute groups such as terpenes and numerous possible solvents, many experiments are necessary to determine the best suitable extraction system. *In silico* prediction of key properties, such as solubility or partition coefficients, helps in gaining essential information and is therefore an advantageous tool to focus and reduce the experimental effort, thereby significantly enhancing process development.

One predictive approach was developed in 1995 by Klamt and co-workers with COSMO-RS (*CO*nductor like *S*creening *MO*del for *R*eal *S*olvents), in which molecules are considered as a cavity in an ideal conductor (dielectric constant *ε* = ∞) [14]. Based on quantum chemical calculation of the 3D structure of a solute and further simulation via statistical thermodynamics, a novel and reliable *ab initio* method for calculating the thermodynamic properties of fluids evolved. Further refinement and parametrization led to an improved model in 1998 [15]. Contrary to empirical methods like the group contribution method, where parameters are fitted to experimental results for different compartments of one molecule, COSMO-RS considers the whole structure at once and determines its chemical potential based on the given structure [16]. In addition to the successful simulation of solvents, prediction of the partition coefficient of a solute in a biphasic system was also achieved with COSMO-RS, with a standard deviation of log*P* ≈ 0.2 [17]. The partition coefficient of a solute can be determined through the reverse proportion of the activity coefficient at infinite dilution in each phase [18]. Nevertheless, the solute behaviour in aqueous solutions could not be simulated properly due to the strong polar forces. This has been improved by considering the hydrogen bonds between two interacting surfaces [19]. COSMO-RS has shown to be a useful tool for solvent screening and prediction of solubility characteristics. Common applications in biotechnology are enhancement of performance in reactive systems [20] and the search for optimal or more selective solvents, especially for natural product extraction [21–23]. In addition, COSMO-RS is used for the screening for alternative solvents in terms of more sustainable, green extractants with reduced environmental impact [24,25]. Thus, *in silico* prediction can reveal promising properties of ionic liquids and deep eutectic solvents in two-phase extraction of hydrophobic compounds [26,27]. Despite the potential in solubility prediction, experimental validation is often required due to the lack of comparable data available for the investigated systems.

With the optimal solvent for the desired application, a suitable process operation can be chosen. Liquid–liquid extraction processes are mostly based on the mixer-settler principle, in which the mass transfer of the solute is achieved by dispersion of two immiscible phases, followed by a settling step for phase separation. Nevertheless, this dispersive technique has disadvantages especially in biotechnological application. The formation of stable emulsions is possible because of the complex aqueous phase composition and damage to the producing host organism can be caused by solvent contact. In addition, the efficiency of batch-wise processing is limited by low product concentrations, making continuous processes more favourable. Continuous liquid–liquid extraction of hydrophobic compounds from an aqueous phase to an organic phase can be performed at the interface of a solid membrane. This membrane-assisted extraction or pertraction, reviewed by several authors [28–30], provides several advantages over the traditional mixer-settler-based extraction. The interphase is stabilized through a microporous membrane, which allows dispersion-free contact of the two phases and therefore prevents emulsion formation. The flow rates of both phases can be chosen independently from each other to reduce the amount of solvent for the extraction. Extraction modules can be built as hollow-fibre contactors, which provide a high interfacial area, and therefore enable high mass transfer and a rapid and efficient extraction process. Hollow-fibre membrane contactors are widely used in different application fields such as gas stripping, removal of metals and contaminants from wastewater, and recovery of valuable compounds from liquids [31,32]. In the latter, the choice of different solvents in combination with hydrophobic and hydrophilic membranes allows selective extraction of the desired substance. Membrane extraction has been successfully applied for the removal of polar components like acids [33,34] as well as aroma compounds [35,36], phenols [37] or terpenes [38] from aqueous solutions or essential oils. Furthermore, membrane-assisted extraction allows its implementation in biotechnological production processes for continuous *in situ* product removal (ISPR), of fermentation or bioconversion products for example [39,40].

**Figure 1.**
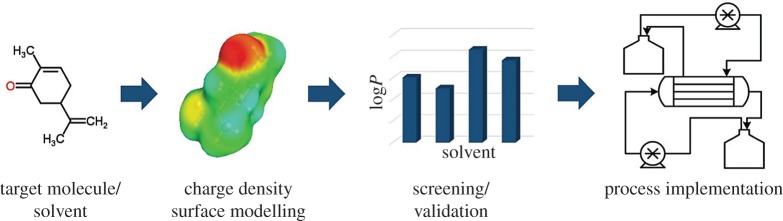
Schematic overview of the integrated workflow. Starting from the initial structural information of the solute, COSMO-RS simulation of the target molecule in various liquid–liquid systems enables the identification of the best suitable solvent for extraction, which can be investigated further in the experimental set-up for process development.

In this study, we demonstrate a procedure for developing a membrane extraction process, beginning with first simulation steps towards the experimental validation and final process integration (figure 1). Two monoterpenes, (S)-(+)-carvone and terpinen-4-ol, were chosen as hydrophobic model substances for potential biotechnological products to be isolated from the aqueous solution. *Ab initio* solvent selection with COSMO-RS was carried out by means of partition coefficients of both substances and simulated log*P* values were compared with those gained by experiments. With the chosen solvent, a membrane-assisted extraction process for one of the model substances was established in a customized small-scale membrane contactor. Two operation modes were evaluated in terms of efficiency, product recovery and their mass transfer, based on the *a priori* determined experimental and simulated partition coefficients. The potential of this process for biotechnological application is discussed and a brief prospect on the process's benefit concerning green separation is given.

## 2. Material and methods

### 2.1. Chemicals

(S)-(+)-carvone and terpinen-4-ol were obtained from Sigma Aldrich, Crailsheim, Germany with a purity of 96% and greater than 98%, respectively. All solvents were purchased in HPLC grade (purity ≥ 95%). Other chemicals were purchased in highest grade from Carl Roth, Karlsruhe, Germany or Sigma Aldrich. Double-distilled water was used in all aqueous systems.

### 2.2. COSMO-RS simulations

Simulations of the partition coefficient for the selected small molecules were based on COSMO-RS (*CO*nductor like *S*creening *MO*del for *R*eal *S*olvents) [14,15]. Here, every component is regarded in a liquid state at an infinite dilution. The chemical potential of the solute molecule is calculated throughout the surface interaction with the surrounding solvent based on quantum chemical equations. 3D structures of (S)-(+)-carvone and terpinen-4-ol were exported from a database (www.chemspider.com, CSID: 15855 and 10756) as initial geometries for further optimization and simulation. *Ab initio* electron structure calculation and primary geometrical optimization were done with the TURBOMOLE v. 7.1 package, where the density functional theory (DFT) has been selected as the underlying method. Further conformation generation was conducted through COSMOconf v. 4.1 [41], in which the optimized structure from TURBOMOLE was taken as an initial conformation. Throughout a multiple and arbitrary conformation generation and selection of the energetically most favourable ones, a bundle of conformations with a triple *ζ* valence electron plus polarization with diffusion function (TZVPD-FINE) parametrization is generated. For each molecule, the resulting *σ*-profiles of a conformation set were implemented into the COSMOtherm v. 17.0 database [42,43]. Water and all organic solvents were taken from the existing COSMO-RS database with the same parametrization. Liquid phase equilibria for the aqueous and organic phases were determined separately and taken for the simulation of the partition coefficient of the solutes in an infinite dilution. Additionally, salt and glycerine concentrations in the liquid were also taken into consideration.

### 2.3. Determination of partition coefficients

Determination of partition coefficients for (S)-(+)-carvone and terpinen-4-ol was performed in capped glass tubes to prevent unspecific adsorption. Terpenes were diluted in 2 ml of water or salt solution (68 mM NaCl, 37 mM NH_4_Cl, 54 mM glycerol; same ionic strength as in M9 minimal cultivation medium) to a final concentration of 0.4 mg ml^−1^. Extraction was carried out with 2 ml of organic solvent for 4 h at 25°C in an Eppendorf Thermomixer. After 10 min of phase separation, samples of both phases were taken and analysed by HPLC. All experiments were performed in triplicate. The partition coefficient *P* was determined with the solute concentrations *c*_s_ and *c*_w_ (equation (2.1)).

2.1 $$P=\frac{c_{s}}{c_{w}}.$$

### 2.4. Membrane-assisted extraction

**Figure 2.**
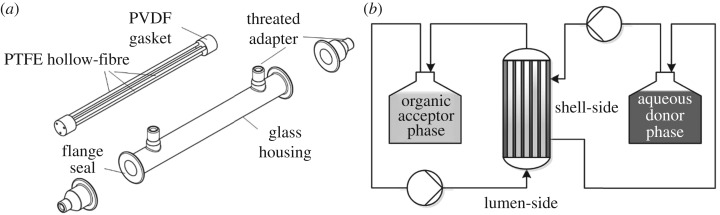
(*a*) Membrane contactor (Memo3 GmbH, Switzerland) with 3 PTFE hollow-fibre (1 µm, 20 cm, 3 mm ID, 56 cm^2^ area) and glass housing. (*b*) Schematic set-up for membrane-assisted extraction of a terpene substance from aqueous phase (shell-side) into the organic n-heptane phase (lumen-side).

Experiments for membrane-assisted extraction of (S)-(+)-carvone were conducted in a customized hollow-fibre membrane contactor module from Memo3 GmbH, Moehlin, Switzerland (figure 2*a*). The module consists of three PTFE membranes with 1 µm nominal pore size, 3 mm ID, 20 cm length and a total inner surface area of 56 cm^2^ (for detailed specifications see appendix, table 5). The membranes are clamped in PVDF gaskets and embedded in a glass housing.

For continuous extraction, an aqueous donor stream and an organic acceptor stream were pumped in counter-current mode through the extraction module in a circuit. The aqueous reservoir contained 0.15 mg ml^−1^ (S)-(+)-carvone in water and the organic reservoir n-heptane (figure 2*b*). PTFE tubing was used in the entire set-up to minimize unspecific adsorption of the hydrophobic (S)-(+)-carvone. Both streams could be pumped through the membrane lumen or the module shell. Pump flow was 3 ml min^−1^ for the lumen-side stream and 40 ml min^−1^ for the shell-side stream. Samples for (S)-(+)-carvone determination were taken at the inlet and outlet of the extraction module for both streams and analysed by HPLC over 8 h of process time. For both modes, the ratio of aqueous to organic phase volume was 2 : 1.

### 2.5. Calculation of mass transfer coefficients and extraction efficiency

Overall mass transfer coefficients *K* for the extraction of (S)-(+)-carvone were calculated in order to compare operational modes in the membrane contactor. For calculations, the resistance-in-series model, where each boundary layer contributes to the diffusive mass transfer, was applied. For a hydrophobic membrane, the mass transfer on the aqueous side *K*_w_ can be calculated with the equation for different modes (equation (2.2)) [44].

2.2 $$\frac{1}{K_{w}d_{i/o}}=\frac{1}{P\;k_{o}d_{o/i}}+\frac{1}{P\;k_{m}d_{\mathrm{lm}}}+\frac{1}{k_{w}d_{i/o}}.$$

*P* is the partition coefficient of the solute, and *K*_w_, *K*_o_ and *k*_m_ represent the individual mass transfer coefficients of the aqueous, organic and membrane boundary layer, respectively. The inner and outer diameter *d*_i_ and *d*_o_ depend on the operation mode with the aqueous phase in the fibre lumen and in the module shell, respectively. *d*_lm_ is the log-mean diameter of the wetted membrane.

Individual mass transfer coefficients for the organic phase, the aqueous phase and the membrane can be obtained with the general mass transfer correlation (equation (2.3)). Mass transfer, expressed by the Sherwood number (*Sh*), correlates with flow characteristics (Reynolds number, *Re*), the diffusivity in the fluid (Schmidt number, *Sc*), and the flow geometry of the module:

2.3 $$Sh\propto Re^{\alpha }\;Sc^{\beta }\;f(\text{geometry}).$$

The geometry factor depends on the operation mode and the stream. For the flow in the module fibre, the Lévêque equation (2.4) is mostly used to determine the fibre-side mass transfer coefficient *k*_fibre_, as it is reasonably accurate for a wide range of flow characteristics [45].

2.4 $$k_{\mathrm{fibre}}=1.62\;\frac{D}{d_{i}}\;{\left(\frac{d_{i}^{2}\nu }{L\;D}\right)}^{1/3}.$$

*d*_i_ is the inner fibre diameter and *υ* the volumetric flow in the fibre over the module length *L*. The molecular diffusion coefficient *D* of (S)-(+)-carvone in the aqueous and organic phase, respectively, was determined with the Wilke–Chang equation [46].

For the description of the shell-side flow parallel to the fibres, several correlations are proposed, which are summed up by Gabelman & Hwang [28]. Equation (2.5) was used for the shell-side mass transfer coefficient *k*_shell_ in this work as it optimally depicts the present flow characteristics [44].

2.5 $$k_{\mathrm{shell}}=\beta \;\frac{D}{d_{h}}\left[\frac{(1-\theta )d_{h}}{L}\right]Re^{0.6}Sc^{0.33}.$$

The module geometry can be expressed through the diffusion coefficient *D*, the hydrodynamic diameter *d*_h_ and the fibre packing fraction *θ*. The factor *β* was found to be 5.8 for hydrophobic membranes [37].

Mass transfer through the hydrophobic membrane *k*_membrane_ can be described with the following equation [44]:

2.6 $$k_{\mathrm{membrane}}=\frac{2\;\varepsilon \;D}{\tau \;(d_{o}-d_{i})}.$$

The porosity *ε* and tortuosity *τ* are membrane-specific. *D* is the diffusion coefficient of the solute in the wetting phase.

An approach, suggested by D'Elia *et al*. [47] and described in detail by Pierre *et al*. [36] and Sciubba *et al*. [33], was used for the determination of the experimental overall mass transfer coefficients. It is applicable to extraction modules in parallel counter-current flow where both phases are circulated in a reservoir, which does not allow the assumption of a steady-state mass transfer.

2.7 $$\begin{array}{r} \ln\left[\left(1+\frac{1}{R}\right)\frac{c_{w}}{c_{w,0}}-\frac{1}{R}\right]=-\left[\frac{Q_{w}\;\Phi }{V_{w}}\left(1+\frac{1}{R}\right)\right]t, \end{array}$$

2.8 $$\begin{array}{r} \Phi =\frac{1-\text{exp}\left[-\frac{K_{w}A}{Q_{w}}\;\left(1-\frac{1}{E}\right)\right]}{1-\frac{1}{E}\;\text{exp}\left[-\frac{K_{w}A}{Q_{w}}\;\left(1-\frac{1}{E}\right)\right]}, \end{array}$$

2.9 $$\begin{array}{r} E=\frac{P\;Q_{s}}{Q_{w}} \end{array}$$

2.10 $$\begin{array}{r} \text{and}\;\;\;R=\frac{P\;V_{s}}{V_{w}}. \end{array}$$

*Φ* is the extraction efficiency in the module with the interfacial area *A*. The factor *E* (equation (2.9)) describes the extraction in the module which depends on the flow rates of the phases *Q*_s_ and *Q*_w_. The extraction factor *R* (equation (2.10)) takes both reservoir volumes *V*_s_ and *V*_w_ into account. With the experimentally determined solute concentrations, extraction efficiency and mass transfer coefficients were obtained by plotting the left side of the equation (2.7) over process time. For a linear behaviour, the slope depends on the extraction efficiency, and the overall mass transfer *K*_w_ for a given aqueous flow *Q*_w_ can be calculated with equation (2.8).

### 2.6. HPLC analysis

Detection and quantification of (S)-(+)-carvone and terpinen-4-ol, in both the aqueous and organic phase, were carried out with a 5 µm Kinetex EVO C18 core-shell column (Phenomenex Ltd, Aschaffenburg, Germany) on an Agilent 1100 HPLC system (Agilent Technologies, Waldbronn, Germany). Mobile phases consisted of water with 20 mM TFA (A) and 90% acetonitrile with 20 mM TFA (B). The elution profile was 35% mobile phase B for 5 min, followed by 45% B for 5 min and another 5 min of equilibration with 35% of B. Mobile phase flow was 0.5 ml min^−1^ at 30°C and the sample injection volume was 5 µl. Terpinen-4-ol and (S)-(+)-carvone were detected at 210 nm and 238 nm, respectively, and quantified with an external calibration curve.

## 3. Results and discussion

### 3.1. Solvent selection with COSMO-RS

For all extraction systems, a suitable solvent in terms of selectivity, partition coefficient, miscibility and others must be chosen. Screening for solvents can be a time- and cost-intensive process when conducted experimentally. Computational prediction of suitable solvents can minimize these drawbacks, but for a reliable application, prediction accuracy must first be ensured. For the extraction, two model substances from the monoterpene class were chosen. Both, (S)-(+)-carvone and terpinen-4-ol are commercially available and can be quantified by HPLC. Nevertheless, they have different functional groups and thus differ in their physico-chemical properties like hydrophobicity, which might affect their predictability.

**Figure 3.**
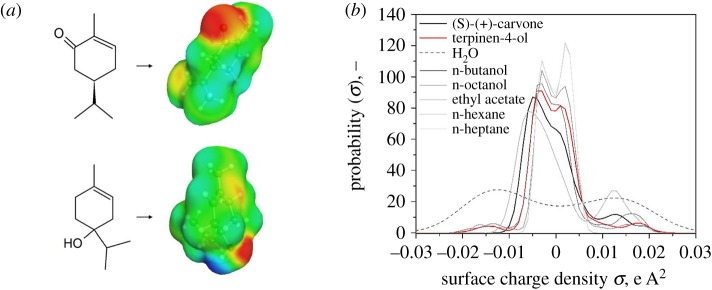
(*a*) Modelling of charge densities (Sigma-surface) of (S)-(+)-carvone (above) and terpinen-4-ol (below) with COSMO-RS. Blue areas indicate positive partial charges, red areas indicate a negative partial charge. Green areas are neutral. (*b*) Probability functions of the surface charge densities, so-called sigma-profiles for the investigated solvents, (S)-(+)-carvone and terpinen-4-ol.

Solvent prediction accuracy was evaluated in terms of the partition coefficient (log*P*) with a set of pure solvents chosen in advance. These include the strictly non-polar solvents n-hexane and n-heptane as well as the slightly more polar solvents ethyl acetate, n-butanol and n-octanol. All solvents form a two-phase system with water and are widely used in liquid–liquid extraction. Mixtures of these solvents were excluded in this study, as they would significantly increase the experimental effort for the comparison of partition coefficients. The model substances were simulated with COSMO-RS and their solubility in the solvents was determined. For that, the computational effort can be reduced significantly by separating the extensive conformational simulation from the statistical thermodynamics. Generated charge density profiles (figure 3*a*) show the three-dimensional structure of the solutes and the partial charge on a displayed surface. The transformed function of the surface charge gives the charge density distribution, the so-called *σ*-profile (figure 3*b*) which also includes parameters such as solute size and cavities. Therefore, conformational simulations have to be conducted only once, and the resulting *σ*-profiles build the dataset for every COSMOtherm simulation. Comparing the *σ*-profiles of the solutes (S)-(+)-carvone and terpinen-4-ol with the organic solvents chosen for liquid extraction, can already provide information on the degree of hydrophobicity beforehand as well as information on polarity and its distribution. The distribution of n-hexane and n-heptane shows a single concentration of the charge density around 0, which indicates non-polar components. Distributions ranging from −0.01 to 0.01 are considered non-polar. The sections from 0.01 to 0.03 and −0.03 to −0.01 show positive and negative screening charges, respectively. Apart from all hydrophobic compounds in figure 3*b*, pure water has a very polar distribution with the indication of a dipolar behaviour, due to the balanced polar parts. Higher similarity in *σ*-profiles results in higher solubility of the solutes. Both (S)-(+)-carvone and terpinen-4-ol are hydrophobic molecules with a small part being partially polar, which means that hydrophobic solvents are more suitable for the liquid extraction of these solutes.

**Figure 4.**
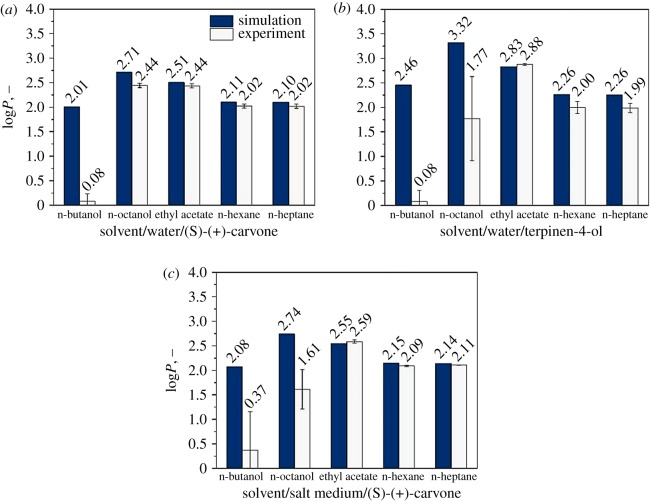
Comparison of simulated and experimental partition coefficients for (S)-(+)-carvone and terpinen-4-ol. (*a*) (S)-(+)-carvone in two-phase system solvent/water. (*b*) Terpinen-4-ol in two-phase system solvent/water. (*c*) (S)-(+)-carvone in two-phase system solvent/salt medium. Error bars represent deviations from technical and analytical triplicates.

Furthermore, COSMOtherm can calculate the relative solubility of solutes in given solvents as the logarithmic solubility of mole fractions. The log*P* values thus obtained can then be validated with experimental data. Figure 4 shows log*P* values for the solutes (S)-(+)-carvone and terpinen-4-ol in the different solvents. For (S)-(+)-carvone, the experimental and simulated log*P* values are in good agreement with the solvents n-heptane, n-hexane, ethyl acetate and n-octanol (figure 4*a*). Deviations are within 0.2 log values, as proposed by Klamt *et al*. [17]. No reasonable comparison could be done with n-butanol as solvents. The short-chain alcohol n-butanol has a significant solubility in water, which makes calculations and the experimentally determination of solute partition behaviour difficult. Even though COSMO-RS takes this partial miscibility in terms of prior calculated phase equilibria into account, log*P* values cannot be considered reliable. This could be explained by the solvent's spatial orientation, which is not being considered in COSMO-RS. Therefore, dipolar molecules, such as water and n-butanol, cannot be simulated with a great accuracy. On the experimental side, (S)-(+)-carvone seems to have just a slightly better solubility in n-butanol than in the aqueous phase. For the partition coefficient of terpinen-4-ol, similar observations were made (figure 4*b*). Log*P* values in n-hexane, n-heptane and ethyl acetate can be predicted with reasonable accuracy. However, COSMO-RS overestimated the log*P* values for terpinen-4-ol as the experimental values are more or less the same as for (S)-(+)-carvone. In addition to the same poor predictability with n-butanol as the organic phase, n-octanol also showed significant deviations in the experimental and calculated partition coefficient. It should be noted that it is not possible to distinguish whether this is due to a poor predictability from COSMO-RS or a result of experimental error.

In many extraction systems, the compound of interest is not dissolved in water but in more complex media, containing salts and other components. Biotechnologically produced hydrophobic substances, such as terpenes, hormones and bioconversion products, in particular, must often be extracted from such complex media. The medium components affect the solubility of the desired substances and therefore on the extraction of these components. Hence, the prediction accuracy of COSMO-RS for the log*P* values of (S)-(+)-carvone in salt medium was evaluated. The salt medium comprised Na^+^, ${\text{NH}}_{4}{\;}^{+}$ and Cl^−^ ions as well as glycerol in concentrations which are used e.g. in microbial cultivation. Comparison with experimentally determined log*P* showed the same tendencies as discussed before, but with slightly higher values that indicate a better solubility of (S)-(+)-carvone in the solvent in the presence of salts and glycerol (figure 4*c*). Noteworthy is once again the significant decrease of the experimental log*P* value for (S)-(+)-carvone in n-octanol and the medium containing salt and glycerol. This could be explained by a strong change in the phase composition due to the very polar n-octanol in combination with glycerol. Because of this, COSMO-RS can be used to predict partition coefficients of hydrophobic substances with good accuracy for non-polar solvents, even in the presence of media components. Besides calculations with the pure solvent components, COSMO-RS can be used with solvent mixtures. The addition of a polar component like n-butanol or ethyl acetate to a hydrophobic solvent such as n-heptane could increase the partition coefficient and provide an enhanced extraction efficiency. Simulations have indicated that a mixture of n-heptane and the alcohol n-butanol increase the solubility of terpinen-4-ol (not shown). Solvent mixtures can be taken into account when process optimization is required or different solutes have to be separated and more product-specific solvent properties are necessary.

When it comes to the choice of solvent, other parameters, such as viscosity, vapour pressure and the solvent toxicity class, must also be considered (table 1). For the membrane extraction process investigated here, the pure solvent n-heptane was chosen due to a high partition coefficient for (S)-(+)-carvone, calculated with COSMO-RS and validated experimentally. Additionally, the lower vapour pressure and a better toxicity classification make n-heptane more favourable than n-hexane or ethyl acetate with similar or even higher partition coefficients.

**Table 1.** Parameters of the evaluated solvents.

	molecular weight, g mol^−1^	viscosity, mPa s	density, kg m^−3^	vapour pressure, hPa^a^	toxicity class^b^
n-butanol	72.1	3.10	0.808	18.3	3
n-octanol	130.2	7.40	0.827	8.7	3
ethyl acetate	88.1	0.43	0.894	97	3
n-hexane	86.2	0.29	0.655	160	2
n-heptane	100.2	0.39	0.684	48	3

^a^According to Reichardt [48].

^b^FDA Guidance for Industry Q3C.

### 3.2. Membrane extraction of (S)-(+)-carvone

Both terpene model substances could be used in a membrane extraction process due to their high partition coefficients. Nevertheless, (S)-(+)-carvone was chosen as it is more stable in an aqueous solution and provides more robust analytics. With n-heptane as a suitable solvent, the extraction of (S)-(+)-carvone was performed in a customized and fully solvent-resistant small-scale membrane contactor. PTFE fibres were clamped in a PVDF block to avoid the need for potting material like epoxy which is prone to damage in the presence of certain solvents. For the same reason, glass replaced conventional polypropylene- or polycarbonate-based housing. The use of only three fibres allows slower lumen-side streams without phase breakthroughs, which can be advantageous when used with biotechnological fluids. A higher interfacial area can be easily provided with additional fibres or parallel modules.

**Figure 5.**
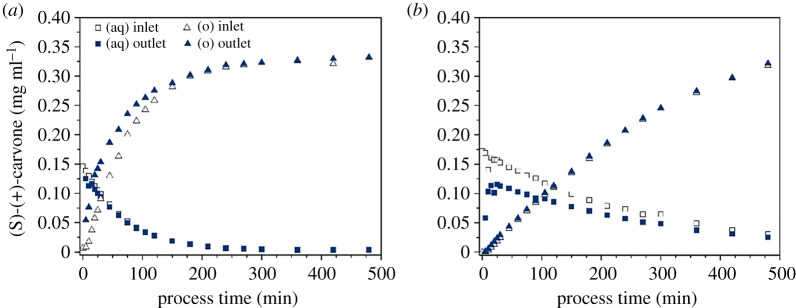
Membrane extraction of (S)-(+)-carvone from aqueous phase with n-heptane over process time. Shown are concentrations at the inlet and outlet of the membrane contactor for both aqueous (aq) and organic (o) streams. Two operation modes with the aqueous donor phase in (*a*) the module shell and (*b*) the fibre lumen. Flows were 3 ml min^−1^ for lumen-side stream and 40 ml min^−1^ for shell-side stream in counter-current. Phase ratio of the aqueous and organic phase was 2 : 1.

Extraction of the solute was carried out in two different operation modes, in which the solute-enriched aqueous donor phase was either in the fibre lumen (in-out mode) or in the shell of the membrane module (out-in mode). The purpose was to compare the two modes in terms of mass transfer and extraction efficiency, as both can be implemented in a continuous recovery process. Initial (S)-(+)-carvone concentrations in the aqueous phase were approximately 0.15 mg ml^−1^, which is the same order of magnitude as the achieved concentrations of microbially produced terpenes [6,7]. In both modes, stable streams without phase breakthrough were established. Transmembrane pressure was kept under the breakthrough threshold of 0.2 bar and (S)-(+)-carvone concentration was monitored over 8 h in both phases at the inlet and outlet. Figure 5*a* shows the solute concentrations with the feed stream in the module shell and n-heptane in the fibre lumen. As expected, (S)-(+)-carvone concentration in the aqueous phase decreases over process time, reaching nearly zero as a consequence of the high partition coefficient, whereas it is enriched in the circulating organic phase. Both the increase and decrease show a linear trend in the first 60 min, during which a constant mass flow can be assumed. Differences in the concentration at the inlet and outlet of the module could only be detected for the tube-side stream, as the throughput in the shell stream was too high. (S)-(+)-carvone transfer in the organic phase was generally lower in the in-out mode, where the equilibrium could not be reached within the 8 h process compared to the out-in mode with a nearly complete extraction after approximately 5 h (figure 5*b*). The fast and complete depletion of the solute in the aqueous phase makes the extraction in the in-out mode ideal for a first recovery step of a biotechnological process, in which the product is harvested from an aqueous solution like a cultivation medium. Especially as no settling time for the phase separation is necessary.

Table 2 compares the overall mass transfer coefficients based on the aqueous stream for both modes. As already indicated by the experimental data given above, (S)-(+)-carvone mass transfer *K*_w_ seems higher for the out-in mode than for the in-out mode. This can be also seen with the calculated *K*_w_ value, which is 4.5 × 10^−6^ m s^−1^ in the out-in mode and therefore twice as high as in the out-in mode with 2.1 × 10^−6^ m s^−1^. For both, the previously COSMO-RS simulated partition coefficients of (S)-(+)-carvone were used. Mass transfer mostly depends on the interfacial area between the two phases, which is higher for the wetting organic phase in the fibre lumen and the feed phase in the module shell. Mass transfer coefficients were also determined with the gained experimental data and the experimental partition coefficient. Equation (2.7), as proposed by D'Elia *et al*. [47], was applied as both phases were recycled in their reservoirs and therefore, with a changing driving force, a steady state cannot be assumed. The experimentally determined *K*_w_ for the in-out mode fits well with the calculated value. For the out-in mode, *K*_w_ is significantly higher when experimentally determined than when calculated, but still tends to be higher than in the in-out mode. However, higher experimental *K*_w_ are unusual, because for low-phase flow rates and Reynolds numbers, the calculated *K*_w_ is normally overestimated with the applied Lévêque solution and therefore higher than the experimentally determined overall mass transfer [28,36]. If that is the case, the more general Graetz solution (Graetz number, *Gz*), which describes forced convection at a low laminar flow, is often applied. Here, the dimensionless Graetz number *Gz* is 0.71 for the tube side flow, whereas the threshold for accurate prediction with the Lévêque solution is *Gz* > 4 [28]. With that, one can assume that the calculated coefficients might even be lower and the difference to the experimental values higher. Another possible explanation for the deviation of the *K*_w_ values might be an insufficient accuracy of the calculated *K*_w_ as a consequence of the low shell-side Reynolds number in combination with the fibre packing fraction, both at the lower limit as proposed by Prasad *et al*. [44]. It is known that the shell-side mass transfer is not fully understood and difficult to predict, as it is highly dependent on the geometry of the module [28]. In addition, the structure of the liquid–liquid interface, which is influenced by the wetting behaviour of the material, might not be accurately considered. *K*_w_ values are in the same or one order of magnitude lower, when compared with the values shown in the literature. Dupuy *et al*. [38] determined mass transfer coefficients of 6.4 × 10^−6^ to 9.5 × 10^−6^ m s^−1^ and 4 × 10^−5^ m s^−1^ for the essential oil monoterpenes citral and limonene, respectively. Pierre *et al*. [36] found *K*_w_ values of 5 × 10^−6^ to 3 × 10^−5^ m s^−1^ for the extraction of sulfur aroma compounds from an aqueous solution with hexane. In both examples, a commercial membrane contactor module was used with an approximate interfacial area of 1 m^2^, which might explain the higher mass transfer coefficients.

**Table 2.** Extraction efficiency, experimentally determined and calculated overall mass transfer coefficients *K*_w_ based on the aqueous stream and the contribution of the individual mass transfer resistances.

	out-in mode	in-out mode
*K*_w_ (experimental), m s^−1^	5.9 × 10^−6^	2.2 × 10^−6^
*K*_w_ (calculated), m s^−1^	4.5 × 10^−6^	2.1 × 10^−6^
efficiency *Φ*, %^a^	5.0	22.2

^a^According to Sciubba *et al*. [33].

**Figure 6.**
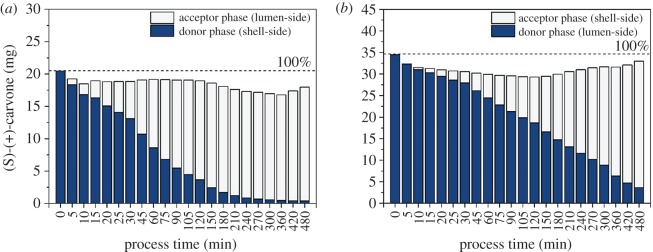
Recovery of (S)-(+)-carvone in the aqueous solution and in n-heptane by membrane-assisted extraction. Shown is the total recovery in both phases for two operation modes: (*a*) aqueous donor phase in module shell, organic acceptor phase in fibre lumen (out-in mode); (*b*) aqueous donor phase in fibre lumen, organic acceptor phase in module shell (in-out mode). Flows were 3 ml min^−1^ for lumen-side stream and 40 ml min^−1^ for shell-side stream in counter-current. Phase ratio of the aqueous and organic phase was 2 : 1.

Several aspects must be considered when evaluating the membrane-assisted extraction as a capture and purification process. One is the extraction efficiency, in which mass transfer is set in relation with the volumetric flow rates of both streams. Both modes differ in their extraction efficiency as defined by Sciubba *et al*. [33]. Despite better mass transfer, extraction efficiency *Φ* with the aqueous phase in the module shell is only 5% compared to 22% with the solute in the lumen of the contactor (table 2). Efficiencies found in the literature range from 2–5% for the extraction of vanillin form aqueous solution with butyl acetate [33] to 54% for different aroma compounds with hexane [35]. With a constant partition coefficient, extraction efficiency mainly depends on the flow ratio of the aqueous and organic phase [49]. It decreases with high aqueous flow rates as applied for the shell side in the studied out-in mode. Consequently, a slower aqueous stream in the fibre lumen might be advantageous if high extraction efficiency is desired. In addition, product recovery plays an important role. Product loss during the process, which can be caused, for example, by the non-specific adsorption of the compound of interest to system components, should be minimized. In particular, hydrophobic substances in aqueous solution like the used (S)-(+)-carvone tend to bind to different synthetic materials used, e.g. in tubing, and therefore lower the recovery and process efficiency. Figure 6 shows the recovery of (S)-(+)-carvone during the process for both modes. For the membrane extraction in out-in mode, approximately 91% of the initial amount of solute could be found in the aqueous and organic phase and 93% for the in-out mode. Furthermore, a concentration factor of approximately 2 was achieved for both modes with the chosen phase ratio. In the organic phase, 98% and 87% of the (S)-(+)-carvone were enriched after 8 h, respectively. Deviation to a complete recovery can be caused, to a small extent, by non-specific adsorbed solute and limited solute determination at low concentrations. Nevertheless, a good overall recovery was achieved in both modes which is a crucial requirement for an efficient purification process.

In the context of a biotechnological application, which is the field this study offers preliminary knowledge for, the evaluated membrane-assisted extraction has the potential to solve specific tasks in the purification of hydrophobic compounds. The customized membrane contactor module allows a fast and nearly complete recovery of (S)-(+)-carvone when operated in the in-out mode. This is advantageous for capture steps in biotechnological processes in which the compound of interest must be removed from an aqueous medium such as a fermentation broth within a reasonable processing time. A higher shell-side feed stream provides better mass transfer and higher throughput, at the costs of lowered extraction efficiency. Hence, it can be considered as an alternative for batch-wise mixer-settler extractions. Additionally, the in-out mode is a good choice for continuous and extended recovery where a uniform feed stream at low flow rates is necessary. This is e.g. the case for shear sensitive liquids such as cell suspensions in an integrated recovery process, in which the product is continuously removed from an aqueous feed medium outside of the reactor (in-stream). Implementation of a membrane-assisted extraction has been applied for several substances such as butyric acid [50], phenolic aroma compounds [51] or isovaleraldehyde [52] but, to the best of our knowledge, not for the secondary metabolites class of terpenes.

In addition, the integrated membrane-assisted extraction has some benefits regarding a more sustainable, green extraction process. This can be achieved, for instance, by process intensification, the reduction of energy consumption and the possibility of using green solvents with reduced environmental impact [53]. Membrane techniques are ideal for process intensification as they show higher efficiency than conventional unit operations in many applications and are easier to integrate [54]. As shown, the here evaluated module can be used for such integrated extraction applications in a lab-scale process, as it provides stability, a reasonable efficiency and scalability through additional fibres. With diffusion as the main driving force for the mass transfer of the hydrophobic compounds, no additional and excessive energy input, like heating in solid–liquid extraction or high pressure in supercritical CO_2_ extraction, is necessary to maintain the process and recover the products. Additionally, the mechanical input is considered lower than in other green extraction techniques such as microwave-assisted extraction and ultrasound-assisted extraction. Compared with the traditional mixer-settler extraction, membrane contactors need significantly reduced hold-up volumes for solvents and even further solvent reduction can be achieved, as both the aqueous and the organic stream as well as their volume ratio can be adjusted independently from each other. Furthermore, the omission of mixing and consecutive settling of two phases in the membrane-assisted extraction extends the application to further, emulsion-forming solvents. As mentioned in the Introduction section, the use of alternative or green solvents with a better HSE (health, safety, environment) profile, is also conceivable for membrane-assisted extraction. Nevertheless, the use of e.g. ionic liquids and deep eutectic solvents in this application as alternatives to petrol-based organic solvents, such as heptane, hexane, alcohols and others, is yet limited. Only a few of these hydrophobic solvents are used in liquid–liquid extraction so far [11,55,56], and still there are drawbacks like mutual miscibility in aqueous/solvent two-phase systems as well as the product isolation from the alternative solvents, which might require additional purification steps.

## 4. Conclusion

In this work, an extraction process for the monoterpene (S)-carvone with a hollow-fibre membrane contactor was successfully developed and applied. Suitable solvents for the extraction were screened in terms of partition coefficients *ab initio* with the predictive tool COSMO-RS for the monoterpenes (S)-(+)-carvone and terpinen-4-ol. Both terpenes were chosen as model substances for biotechnologically produced hydrophobic compounds which the developed process is meant to address. Simulated and experimental partition coefficients were compared and found to be in good accordance for the non-polar solvents ethyl acetate, n-hexane and n-heptane. However, for the more polar solvents n-butanol and n-octanol, the agreement of simulation and experiment was poor due to the partial miscibility of the solvents with water. COSMO-RS can also be used for solubility prediction in liquid–liquid systems in the presence of salts and glycerol which are typical medium components in biotechnological processes. Owing to its lower toxicity and the high partition coefficient, n-heptane was chosen as a solvent for the membrane extraction process of (S)-(+)-carvone from aqueous solution. A customized and fully solvent-resistant small-scale contactor was applied for extraction and two operation modes were tested with the aqueous feed phase in the fibre lumen (in-out mode) and the module shell (out-in mode). The latter shows better mass transfer and a nearly complete extraction of (S)-(+)-carvone within 8** **h of process time, but extraction efficiency was lower compared to the feed phase being in the fibre lumen. The prior COSMO-RS simulated and experimentally determined partition coefficients were used to calculate and compare the mass transfer in both operation modes. An overall product recovery greater than 90% and a concentration factor of 2 were obtained making them applicable for fast batch-wise product capture or in continuous and integrated product removal. The herein presented process with the combination of *in silico* prediction supported solvent screening and extraction in a hollow-fibre membrane contactor is found to be a promising approach for the recovery of valuable hydrophobic substances like terpenes from biotechnological solutions and is easily transferable to other compounds. Despite the herein-used COSMO-RS, other predictive tools can also be considered for solvent properties prediction. Nevertheless, a more accurate prediction in polar solvents and better description of mass transfer kinetics in the membrane contactor at low flow rates remain as challenging topics for the future.

## Data accessibility

The datasets supporting this article are accessible from the Dryad Digital Repository: https://doi.org/10.5061/dryad.b3cf6 [57].

## Appendix

See tables 3–5.

**Table 3.** Nomenclature.

*A*	interfacial area	m^2^
*c*	concentration	mg ml^−1^
*D*	molecular diffusion coefficient	m^2^ s^−1^
*d*	diameter	m
*f*	factor	—
*Gz*	Graetz number	—
*k*	individual mass transfer coefficient	m s^−1^
*K*	overall mass transfer coefficient	m s^−1^
*L*	length	m
*P*	partition coefficient	—
*Q*	flow rate	m^3^ s^−1^
*Re*	Reynolds number	—
*Sc*	Schmidt number	—
*Sh*	Sherwood number	—
*t*	time	s
*V*	volume	m^3^
*subscripts*		
*h*	hydrodynamic	
*i*	inner	
*lm*	log mean	
*m*	membrane	
*o*	outer	
*s*	solvent, organic	
*w*	water, aqueous	
*0*	initial	
*Greek symbols*		
*α*	coefficient (equation (2.2))	—
*β*	coefficient (equation (2.2))	—
*ε*	porosity	—
*θ*	fibre packing fraction	—
*σ*	surface charge density	e Å^2^
*τ*	tortuosity	—
*ν*	flow velocity	m s^−1^
*Φ*	extraction efficiency	—

**Table 4.** Diffusion coefficients of (S)-(+)-carvone at 25°C calculated with the Wilke–Chang equation [46] (molar volume at boiling point: 159.8 cm^3^ mol^−1^).

in water, cm^2^ s^−1^	8.1 × 10^−5^
in n-heptane, cm^2^ s^−1^	2.6 × 10^−6^

**Table 5.** Characteristics of the membrane contactor module.

*module*	
length, cm	20
diameter, cm	2.0
*fibre*	
number	3
inner diameter, mm	3.0
outer diameter, mm	3.1
nominal pore size, μm	1.0
porosity	0.55^a^
tortuosity	2^b^
total inner area, cm^2^	56.5
total outer area, cm^2^	58.4

^a^According to Memo3 GmbH.

^b^Estimated [28].
